# Microparticle Hydrogel Material Properties Emerge from Mixing-Induced Homogenization in a Poly(ethylene glycol) and Dextran Aqueous Two-Phase System

**DOI:** 10.1021/acs.macromol.3c00557

**Published:** 2023-10-30

**Authors:** Thomas J. Tigner, Grant Scull, Ashley C. Brown, Daniel L. Alge

**Affiliations:** † Department of Biomedical Engineering, Texas A&M University, College of Engineering, College Station, Texas 77845, United States; ‡ Joint Department of Biomedical Engineering, North Carolina State University and University of North Carolina at Chapel Hill, College of Engineering, Raleigh, North Carolina 27695, United States; § Comparative Medicine Institute, North Carolina State University, Raleigh, North Carolina 27695, United States; ∥ Department of Material Science and Engineering, Texas A&M University, College of Engineering, College Station, Texas 77845, United States

## Abstract

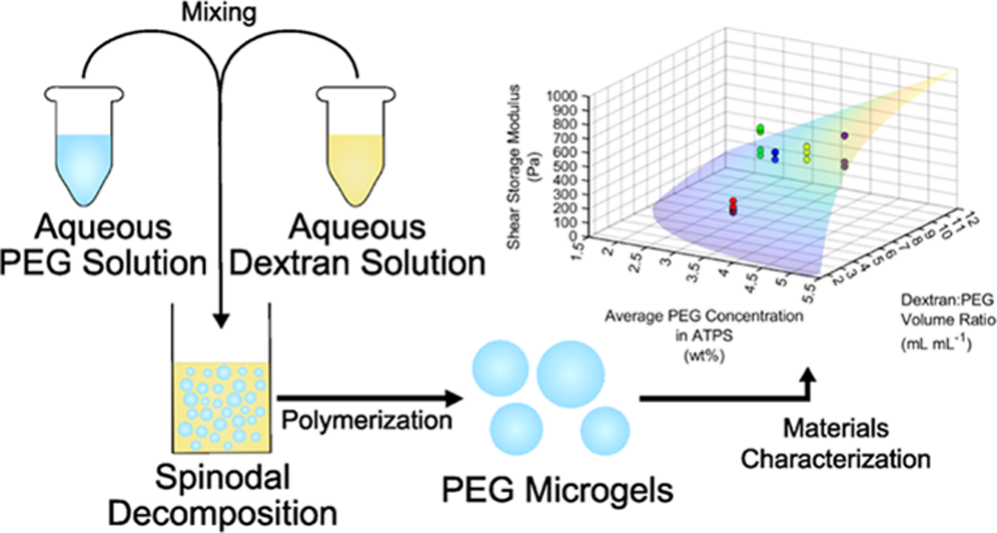

Polymer–polymer aqueous two-phase systems (ATPSs) are attractive for microgel synthesis, but given the complexity of phase separation, predicting microgel material properties from ATPS formulations is not trivial. The objective of this study was to determine how the phase diagram of a poly(ethylene glycol) (PEG) and dextran ATPS is related to the material properties of PEG microgel products. PEG-dextran ATPSs were prepared from four-arm 20 kDa PEG-norbornene and 40 kDa dextran in phosphate buffered saline (PBS), and the phase diagram was constructed. PEG microgels were synthesized from five ATPS formulations using an oligopeptide cross-linker and thiol-norbornene photochemistry. Thermogravimetric analysis (TGA) revealed that the polymer concentration of microgel pellets linearly correlates with the average concentration of PEG in the ATPS rather than the separated phase compositions, as determined from the phase diagram. Atomic force microscopy (AFM) and bulk rheology studies demonstrated that the mechanical properties of microgels rely on both the average concentration of PEG in the ATPS and the ATPS volume ratio as determined from the phase diagram. These findings suggest that PEG-dextran ATPSs undergo homogenization upon mixing, which principally determines the material properties of the microgels upon gelation.

## Introduction

Hydrogels are networks of high-cross-linked hydrophilic polymers that swell to many times their dry mass in water. Their versatility and tunability have afforded hydrogels with broad applicability in drug delivery, biosensing, and tissue engineering.^1^ Hydrogels with micron-scale geometries (microgels) are particularly interesting because of their favorable drug release kinetics, routes of hydrogel implantation, and host–implant interactions.^2,3^ Importantly, microgels can be extruded through a needle and delivered by injection, whereas bulk hydrogel slabs require invasive surgery.^3^ In addition, in the field of tissue engineering, microgels have been assembled into bulk scaffolds with inherently interconnected microporosity by packing and in situ covalent-linking or “annealing”. These microporous annealed particle scaffolds have been applied to various *in vitro* cell culture and *in vivo* injury models^3−9^ and have been shown to enhance migration of cells and tissue into the scaffolds relative to nanoporous hydrogel controls.^4−6^ Moreover, microgels can be assembled into films on bulk hydrogel substrates to modulate surface viscoelastic properties for *in vitro* cell culture applications.^10^ Microgels have also been used in three-dimensional (3D) bioprinting. Jammed microgel pellets yield above critical shear stresses and exhibit shear thinning behavior, making them an attractive bioink platform for extrusion bioprinting.^3,11−14^ Cells exhibit high viability in bioprinted constructs whether encapsulated within the microgels of the bioink^11,15^ or mixed with the microgels in the bioink.^12^ The extrusion print parameters, as well as microgel size distribution and stiffness can be tuned to adjust the rheological properties of jammed microgel bioinks and optimize print fidelity and cell viability.^15^ Microgels have also been incorporated in extrudable bioink formulations as viscosity modifiers^16^ and porogens.^17^ High-fidelity prints with resolutions down to 50 μm have also been achieved by microplotting with microgel bioinks.^18^

Microgels can be synthesized through a variety of techniques, broadly classified as micromolding or phase-separating liquid systems. Liquid systems are advantageous because they do not rely on complex fabrication processes to produce a microgel mold and can be easily scaled.^19^ Among liquid systems, water-in-oil (W/O) emulsions are commonly used for microgel synthesis by suspension. However, the products must be extensively washed to remove traces of the cytotoxic surfactant and organic solvent before use in biological applications. In contrast, aqueous two-phase systems (ATPSs) are formed by demixing of two water-soluble species in aqueous solution. Both polymer–salt and polymer–polymer ATPSs have been used extensively to synthesize microgels through fabrication techniques including microfluidics^20,21^ and batch methods.^19^ Importantly, ATPSs are fully aqueous and do not require organic solvents or surfactants.

Poly(ethylene glycol) (PEG) and dextran ATPSs are particularly popular for microgel synthesis. For example, dextran microgels with predictable sizes have been produced from methacrylated-dextran and linear PEG in aqueous solution.^22,23^ PEG microgels have also been synthesized from PEG-dextran ATPSs with a wide variety of chemical modifications. For example, Murphy et al. used a PEG-dextran ATPS to produce oligopeptide-functionalized PEG microgels capable of binding and sustaining the release of vascular endothelial growth factor (VEGF).^24−26^ The peptides were added to the PEG-dextran ATPS during microgel synthesis and mediated VEGF binding and sustained release for up to 30 days.^24^ Released VEGF exhibited functional activity in *in vitro* endothelial cell proliferation assays.^24^ PEG microgels with tunable degradation profiles have also been synthesized from PEG-dextran ATPSs by incorporating hydrolytically degradable ester bonds or MMP-degradable peptide cross-linker sequences into the hydrogel networks.^27^ As another example, our lab has previously used a PEG-dextran ATPS to synthesize PEG microgels by thiol-norbornene click chemistry. The gelation reaction was conducted off stoichiometry such that excess norbornene was left available for subsequent reaction with tetrazine-functionalized bioactive proteins, specifically alkaline phosphatase and glucose oxidase.^28^ Lin et al. also reported the synthesis of PEG microgels from PEG-dextran ATPSs using thiol-norbornene chemistry.^29^

Although these contributions demonstrate the utility of PEG-dextran ATPSs in microgel synthesis and the amenability of the systems to chemical alterations, it is still unclear how ATPS composition influences the material properties of the microgels, such as their polymer concentration and stiffness. Critically, these material properties are unintuitive to estimate because the polymers and solvent are able to partition between the separating phases of the polymer–polymer ATPSs. Consequently, a clear understanding of the thermodynamics of phase separation could be indispensable for understanding microgel material properties.

**Figure 1:**
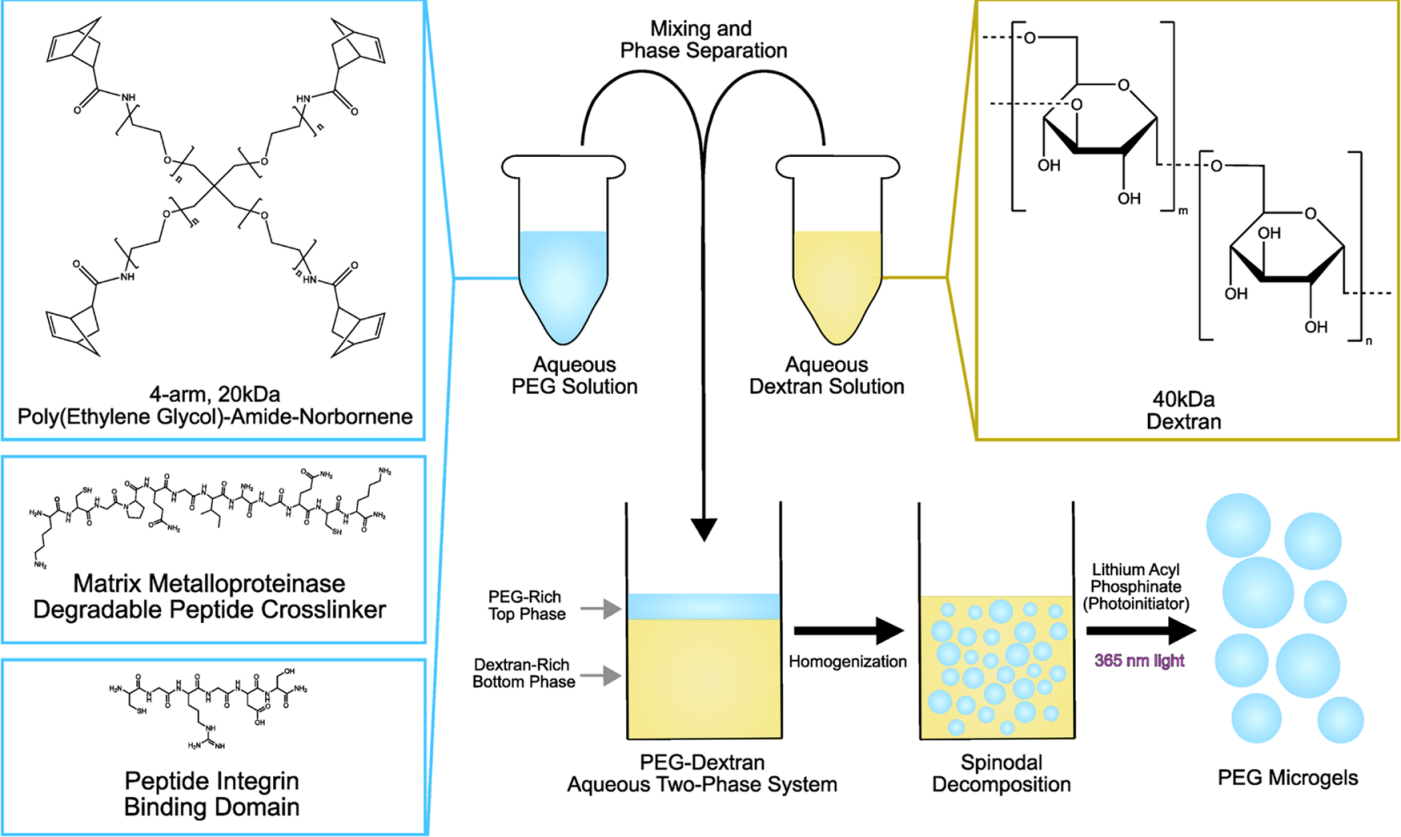
Schematic representation of the process for constituting PEG-dextran ATPSs and synthesizing PEG microgels. An aqueous solution of PEG, cysteine-containing peptides, and photoinitiator was mixed in different amounts with an aqueous solution of dextran. After being mixed, PEG microgels were polymerized in the ATPS by thiol-norbornene photochemistry. The relationship between ATPS composition and microgel material properties was investigated.

Techniques for characterizing phase separation in ATPSs are well established in the field of biochemistry, where ATPSs are extensively applied for the extraction of biomolecules and biological particles.^30^ In these cases, phase diagrams are principally used to describe the dynamics of ATPS separation and to predict the compositions of their separated phases. However, it is not clear whether they are useful for predicting microgel material properties. Where phase diagrams have been applied in microgel synthesis, it is limited to predicting microgel size.^22,23^ Thus, the objective of this study was to determine if PEG microgel properties can be predicted from parameters derived from the phase diagram of a PEG-dextran ATPS, or if other variables, such as the average composition of the ATPS, were more predictive (Figure 1). To this end, we experimentally determined the binodal for an ATPS of four-arm 20 kDa PEG and 40 kDa dextran in phosphate buffered saline (PBS) and developed a hypothesis that the material properties of the microgels would be directly related to the concentration of PEG in the PEG-rich phase as predicted in the phase diagram. By this hypothesis, microgel formulations that were expected to produce microgels of soft, intermediate, and high stiffnesses were identified. We then characterized these microgels on the basis of micromechanical stiffness as well as size and polymer content. The data were then fit to phenomenological models to test our hypothesis and elucidate correlations between ATPS formulation and microgel properties.

## Experimental Section

### Materials

4-arm 20 kDa poly(ethylene glycol)-amine was purchased from JenKem Technology USA (SKU: 4ARM-NH2Cl). 40 kDa Dextran (40 kDa) was purchased from Thermo Scientific (formerly manufactured by Alfa Aesar, Catalog number: J63690.18). Lithium phenyl-2,4,6-trimethylbenzoylphosphinate (lithium acyl phosphinate, LAP) was purchased from Sigma-Aldrich (CAS: 85073-19-4). KCGPQGIAGQCK and CGRGDS peptides were custom-ordered from AAPPTec. (3-Mercaptopropyl)trimethoxysilane was purchased from Sigma-Aldrich (CAS: 4420-74-0). 4-arm 20 kDa poly(ethylene glycol)-thiol (PEG-SH) was purchased from Laysan Bio, Inc. (Item number: 4arm-PEG-SH-20K-1g). All materials were used as received unless otherwise specified.

### Functionalization of PEG Macromonomer

PEG-amide-norbornene (PEGaNB) was functionalized from 4-arm 20 kDa PEG-amine according to previously published materials and protocols, except PEG-amine was substituted for PEG-hydroxyl.^28^ Briefly, norbornene carboxylic acid was reacted with diisopropylcarbodiimide in anhydrous dichloromethane to produce an anhydride. The product was filtered into a round-bottom flask containing a solution of PEG-amine and triethylamine in dichloromethane. The aminolysis reaction was allowed to proceed overnight. The PEGaNB product was then precipitated in ice-cold diethyl ether and left under vacuum for 2 days. The product was reconstituted in deionized (DI) water, dialyzed against DI water for 2 days, frozen, and lyophilized. The functionalization of the product with norbornene groups was confirmed by proton NMR spectroscopy in deuterated water. Two batches of PEGaNB were used in this study, both with functionalizations greater than 93% (Figure S1). The lyophilized product was reconstituted in PBS to a concentration of 20 wt % for use in subsequent experiments.

### Determination of PEG-Dextran ATPS Binodal

The binodal morphology of the PEG-dextran ATPS in PBS was determined in two experiments by cloud-point titration. Briefly, in the first experiment, a 20 wt % solution of PEGaNB was titrated against 500 μL (∼550 mg) of a 42.86 wt % solution of dextran. In the second experiment, a 30 wt % solution of dextran was titrated against 300 μL (∼300 mg) of a 20 wt % solution of PEGaNB. In both experiments, the titrant was added to the analyte in volume increments of 5–30 μL. After each addition, the mixture was vortexed and observed for clouding, which indicated phase separation. If clouding was observed, the composition of the mixture was recorded and PBS was added in 10–150 μL increments until clouding subsided. Titrant was then added in small volume increments until clouding was again observed, and so on. The experiments were ended after at least 850 μL of the titrant solution in total was added to the analyte solution. The ATPS compositions that lay on the binodal were fit by least-squares nonlinear regression to a binodal model (eq 1) previously applied by others^31,32^ with slight alteration. Specifically, an inverse term was applied in this model so that the binodal would be asymptotic with the ordinate. This precluded prediction of negative polymer concentrations on the binodal, which are not physically realizable.
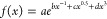
1

### Determination of PEG-Dextran ATPS Tie-Lines

Tie-lines for the PEG-dextran ATPS in PBS were determined by gravimetric phase separation experiments. ATPSs were prepared at 500 mg in total mass in microcentrifuge tubes at six different compositions according to Table 1. Samples were prepared in triplicate for each ATPS formulation. The ATPSs were vortexed until well mixed and then allowed to phase-separate at room temperature for 2 days. After separation, the PEG-rich top phase was decantated from the mixture using a variable volume pipet, and its mass was determined on an analytical balance. The weight of the top phase along with other known ATPS parameters were used to solve a system of equations (eqs S1–S7) including mass balance equations and the previously determined equation for the binodal (a detailed discussion of the system of equations is included in the Supporting Information). The compositions of the separated phases at equilibrium were then calculated and enabled construction of tie-lines.

**Table 1:** ATPS Compositions in Gravimetric Phase Separation Studies

		polymer concentrations in top phase^a^ (wt %)		polymer concentrations in bottom phase^a^ (wt %)	
average PEG concentration (wt %)	average Dextran concentration (wt %)	PEG	Dex	PEG	Dex
5.0	6.0	7.34 ± 0.02	0.91 ± 0.01	0.31 ± 0.01	16.21 ± 0.10
6.0	8.0	10.14 ± 0.45	0.28 ± 0.04	0.09 ± 0.05	19.10 ± 1.32
7.0	10.0	12.85 ± 0.35	0.15 ± 0.01	0.02 ± 0.01	21.78 ± 0.73
8.0	12.0	14.56 ± 0.59	0.12 ± 0.01	6.24 × 10^–4^ ± 4.92 × 10^–4^	26.56 ± 1.36
11.0	18.0	25.97 ± 1.19	0.06 ± 2.41 × 10^–3^	∼0	31.24 ± 1.07
13.0	24.0	35.31 ± 1.51	0.04 ± 1.31 × 10^–3^	∼0	38.00 ± 0.92

a Polymer concentrations were calculated from eqs S1–S7 with the raw data collected in gravimetric phase separation studies. Values are the average ± standard deviation of the replicate samples. Note that the error associated with the binodal was not propagated in these standard deviations. Monte Carlo simulations (Table S2) were conducted to estimate the total error associated with the tie-lines.

#### Dextran

PEG volume ratios were calculated as described elsewhere.^30^ Briefly, they were determined from tie-lines as the ratio of the length of the line connecting the intersection of the tie-line with the binodal near the *y*-axis and the average composition of the ATPS to the length of the line connecting the average composition of the ATPS and the intersection near the *x*-axis. The ratio was corrected for the relative densities of the separated phases. The densities were approximated for the sake of simplicity. The PEG phases were assumed to have a density of 1.0 g mL^–1^, and the dextran phases were assumed to have a density of 1.1 g mL^–1^. Interpolated tie-lines were determined from the empirically determined tie-lines by arc-length continuation of a conjugate curve. The conjugate curve was fit by linear regression to a quadratic model. Monte Carlo simulations with 10,000 iterations were also conducted to assess the error associated with the interpolated tie-lines (Table S3). The simulations agree with the numerical solutions reported in Table 1.

### Microgel Synthesis

Microgels were synthesized from PEG-Dextran ATPSs in PBS at five different formulations, as outlined in Table 2. General microgel sizes in Table 2 were predicted from previous observations that higher continuous phase: disperse phase volume ratios are associated with smaller microgel diameters.^22,23^ The general predicted mechanical properties in Table 2 were hypothesized to be correlated with the concentration of PEG in the PEG-rich phase. All stock solutions were prepared in PBS. The stock solution of 20 wt % PEGaNB was mixed in microcentrifuge tubes with stock solutions of 100 mM KCGPQGIAGQCK peptide, 72.47 mM CGRGDS peptide, 100 mM LAP, and excess PBS in appropriate volumes to achieve the desired ATPS formulation. The PEG-peptide solutions were then added to an appropriate volume of a 45 wt % dextran stock solution in a glass scintillation vial. (Note: the 45 wt % dextran stock solution was too dilute to prepare the stiff microgel formulation, so in this case, the PEG-peptide solution was added to dry dextran powder in a scintillation vial). All microgel formulations were synthesized at 1000 mg of working PBS mass. For all microgel formulations, KCGPQGIAGQCK peptide was added to a working thiol-norbornene ratio of 0.75, CGRGDS peptide was added to a working concentration of 0.1 mM in the ATPS, and LAP was added to a working concentration of 5 mM in the ATPS. The scintillation vial was mixed on a standard vortex mixer at maximum speed until it was well mixed (ca. 1–2 min for all formulations except the stiff formulation, which was mixed for ∼15 min). Unless mentioned otherwise, the scintillation vials were immediately exposed to UV light from a mercury arc lamp (OmniCure S2000) equipped with a 365 nm filter attachment at 50 mW cm^–2^ for 180 s to cross-link the microgels. After polymerization, the mixtures were diluted to 100 mL in total volume in PBS and split between two 50 mL conical tubes. The mixtures were centrifuged at 8000 rcf for 5 min to pellet the microgels, and the supernatants were decanted. The pellets were washed once with 70% ethanol at 20 mL in total volume per conical tube and three more times with DI water at the same volume. The pellets were then resuspended in DI water at the same volume. The microgels were allowed to swell overnight at 4 °C before use. Phase contrast images of the microgel suspensions were captured using a Zeiss Axio Vert.A1, and microgel diameters were manually measured using the “Measure” tool on ImageJ. At least 300 microgels were measured per formulation. The polydispersity indices (PDI) of the microgel diameters were computed for each formulation as the ratio of the variance of the microgel diameters to the square of the number average of the microgel diameters.

**Table 2:** ATPS Formulations for Microgel Synthesis

		polymer concentrations in top phase^a^ (wt %)		polymer concentrations in bottom phase^a^ (wt %)					
average PEG concentration (wt %)	average Dextran concentration (wt %)	PEG	Dex	PEG	Dex	Dextran:PEG volume ratio^a^ (mL mL^–1^)	general predicted microgel size	general predicted mechanical properties	data marker color
3.5	21.65	18.10	0.08	3.01 × 10^–4^	26.82	3.79	NA	soft	red
3.5	30.97	30.00	0.05	∼0	35.05	6.88	medium	intermediate 1 (I.1)	blue
2.2	32.48	29.99	0.05	∼0	35.05	11.48	small	intermediate 2 (I.2)	green
5.3	28.88	30.01	0.05	∼0	35.06	4.24	large	intermediate 3 (I.3)	purple
4.0	39.36	44.75	0.04	∼0	43.22	9.26	NA	stiff	yellow

a Polymer concentrations and volume ratios are calculated from interpolated tie-lines in the phase diagram (Figure 3). Monte Carlo simulations (Table S3) were also conducted to assess the error associated with the numerical values reported above.

For studies pertaining to the influence of coarsening time on microgel material properties, formulation intermediate 1 microgels were allowed to rest for 0, 20, 35, or 60 min following mixing before they were exposed to 365 nm light.

### Thermogravimetric Analysis (TGA) of Packed Microgel Pellets

1 mL of the microgel suspensions was transferred to microcentrifuge tubes. The tubes were centrifuged at 20,000 rcf for 1 h at room temperature. To ensure samples were not compressed during analysis, the microgel pellets were allowed to recover their shape overnight at room temperature. The supernatant was then decanted from the microgel samples. About 10–30 mg of the pellet was transferred to a ceramic dish that was placed on a platinum pan. The sample was then loaded into a TA Instruments Q50 Thermogravimetric Analyzer and subjected to a linear ramp from 40 to 600 °C at 10 °C min^–1^ under a constant flow of nitrogen at 20 mL min^–1^. The concentration of polymer in the packed microgel pellets was determined as the relative weight associated with polymer decomposition in the thermogram (occurring around 350–425 °C) to the total weight of the sample. At least three samples were analyzed per microgel formulation.

### Atomic Force Microscopy (AFM)

Glass coverslips (8 mm diameter) were thiolated using (3-mercaptopropyl)trimethoxysilane. To 170 μL of the reagent was added 30 mL of 200-proof ethanol with two drops of glacial acetic acid. Coverslips were submerged in this solution for about 2 min. They were then rinsed with 200-proof ethanol and placed in an oven at 80 °C for 15 min. Microgels were immobilized on these coverslips for analysis. Immobilization was achieved by mixing the microgel suspensions with LAP to a working concentration of 5 mM. About 100 μL of the microgel suspension in LAP solution was added to the surface of each of the thiolated coverslips, and the coverslips were exposed to 365 nm UV light from an Omnicure S2000 mercury arc lamp at 50 mW cm^–2^ for 180 s. The sample coverslips were left in DI water in individual wells of a six-well plate until use.

Coverslip-immobilized microgels were subjected to AFM contact force mode analysis in DI water using a uniqprobe scanning probe microscopy sensor from NanoAndMore (CP-gp-CONT-PS-A cantilever; S/N: 2021NM096/1) and an Asylum MFP-3D-Bio atomic force microscope (Asylum Research, Oxford Instruments, Goleta, CA) with Igor Pro software. The colloidal particle was polystyrene with a diameter of 1.98 μm ± 10%. Force maps (10 × 10 μm) were collected, and micromechanical stiffnesses were determined for individual microgels by fitting the force maps to the Hertz model and taking the average stiffness value of the force map. At least 9 microgels were analyzed per group.

### Assembly of Microgel Scaffolds

Microgel suspensions were centrifuged at 8000 rcf for 5 min, and the supernatants were decanted. 500 μL of the packed microgel pellets were then transferred to microcentrifuge tubes using a variable volume pipet. The pellets were centrifuged again at 10,000 rcf for 3 min, and any excess supernatant was decanted. This was repeated two more times until no supernatant was observed above the packed microgel pellets. The pellets were adjusted to 250 μL by removing excess volume using a variable volume pipet. Four-arm 20 kDa PEG-thiol (PEG-SH) and LAP were added to the microcentrifuge tubes to 100 μM and 3 mM, respectively. Table S1 shows the estimated ratio of thiols in PEG-SH to excess norbornene in the microgel pellets for each formulation. The microcentrifuge tubes were then centrifuged three more times at 10,000 rcf for 3 min, and any excess supernatant was decanted from the pellets after each centrifugation cycle. The packed pellets were transferred to 8 mm diameter by 4 mm thick cylindrical silicone gasket molds by using plastic scoopulas. The molds were individually exposed to UV light from an Omnicure S2000 with 365 nm filter attachment at 50 mW cm^–2^ for 180 s. After annealing, the microgel scaffolds were transferred to about 8 mL of DI water in individual wells of a 6-well plate. The scaffolds were allowed to equilibrate at room temperature overnight before being used in rheology. Scaffolds were tested within 4 days of assembly.

### Rheology of Microgel Scaffolds

Microgel scaffolds were subjected to frequency and strain sweeps on an Anton Paar MCR 301 rheometer. The scaffolds were transferred to the rheometer stage and blotted with Kimwipes to remove excess DI water. Rheology was conducted by using an 8 mm parallel plate geometry at a gap distance of 3–4 mm. Frequency sweeps were conducted from 0.001 to 10 Hz, ramped logarithmically, at a constant strain of 1%, and data was collected every 2 s at 10 points per decade. Strain sweeps were collected on each microgel scaffold after frequency sweeps. Strain was ramped logarithmically from 0.1 to 500% at a constant frequency of 0.25 Hz. Data was collected every 4 s at 10 points per decade. At least three scaffolds were analyzed by rheology per microgel formulation.

### Light Microscopy of Phase Separation

Formulation intermediate 1 ATPSs were prepared at 3.3 mL in a glass-bottom 30 mm culture dish and homogenized using a sonic probe. Phase separation was captured by using an AmScope T490b microscope. Two ATPSs were prepared for these experiments. The first ATPS was prepared by using only PEG, dextran, and PBS. 30 μL of Trypan blue, a small molecule dye that partitions into the PEG-rich phase, was also added to the ATPS to improve contrast. Phase separation was captured over the course of 60 min following homogenization. The second ATPS was prepared from all of the ATPS components including the polymers, peptides, and photoinitiator. Trypan blue was not added to this ATPS. The process of phase separation was captured over the course of 20 min, and it was allowed to proceed for 5 min following homogenization without additional input. The ATPS was then exposed to UV light from a handheld flashlight (Alonefire SV003 10W 365 nm) for 5 min, and the ATPS was recorded for an additional 10 min following cessation of UV exposure.

### Statistics

For microgel sizing, TGA, AFM, and bulk microgel scaffold rheology experiments, the data sets were initially subjected to a Bartlett’s test for homoscedasticity. If the null hypothesis was not rejected, a one-way analysis of variance (ANOVA) was applied with Tukey’s post hoc analysis for multiple comparisons. If the null hypothesis for Bartlett’s test was rejected, the data sets were subjected to the Kruskal–Wallis test with Dunn test post hoc for multiple comparisons. Critical P-values were set at 0.05 for all of the statistical examinations.

Least-squares method was applied for all regression analyses. For the TGA, AFM, and bulk microgel scaffold rheology experiments, adjusted and predicted *R*^2^ values were also computed to assess overfitting of the data. Variances-covariance matrices are reported for regressions performed (Tables S4–S7).

Monte Carlo simulations were performed using a custom Matlab script to assess the error associated with both empirically determined (Table S2) and interpolated tie-lines (Table S3). 10,000 iterations were performed for each model input. Where applicable, simulations were conducted using raw data, and then replicate error was assessed by averaging simulation outputs and propagating their estimated errors. The results of the simulations were in agreement with the numerical solutions provided in Tables 1and 2.

## Results and Discussion

**Figure 2:**
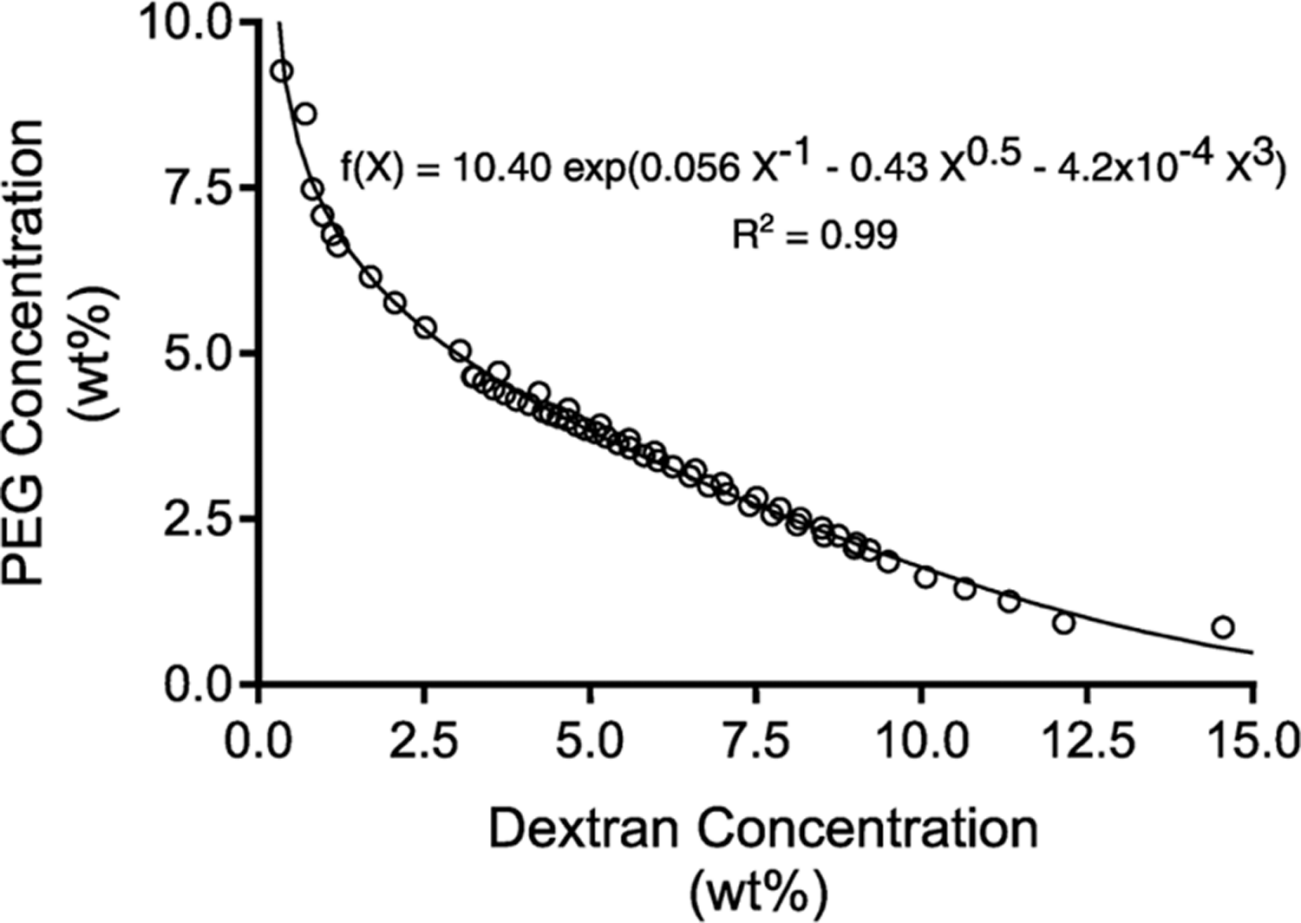
Cloud-point titrations enabled the construction of the binodal for PEG-dextran ATPSs. Empirically determined ATPS compositions that lie on the binodal are represented by open circles. The binodal is represented by the solid black curve.

The objective of this study was to determine if the phase diagram of a PEG-dextran ATPS could be used to predict the properties of PEG microgels. The binodal of PEG-dextran ATPSs was determined by two cloud-point titration experiments, and the raw data was fit to a model (eq 1) previously used by others with little alteration (Figure 2).^31,32^ Cloud-point titrations revealed that phase separation occurs in PEG-dextran ATPSs at working compositions with PEG concentrations greater than 9.3 wt % and dextran concentrations as low as about 0.4 wt %. Moreover, phase separation occurred at dextran concentrations greater than about 14.5 wt % and PEG concentrations as low as about 0.9 wt %. This suggests that the separated phases are practically completely enriched in their respective polymer at average PEG concentrations greater than about 9.3 wt % or average dextran concentrations greater than about 14.5 wt %, regardless of the average concentration of the other polymer.

**Figure 3:**
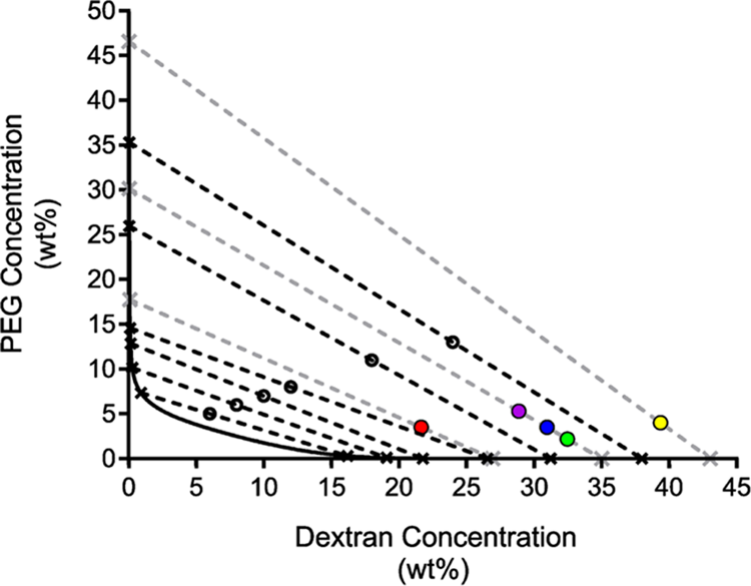
Gravimetric phase separation studies enabled the determination of tie-lines in the phase diagram. Black dashed lines represent empirically determined tie-lines with average ATPS compositions marked with open circles. Compositions of their separated phases are marked with an *x* where the tie-lines intersect the binodal, represented by the solid black curve. Gray dashed lines represent interpolated tie-lines for the five ATPS formulations used in microgel synthesis with average compositions marked with colored circles. Data marker colors correspond to microgel formulations as follows: Red: soft, blue: intermediate 1, green: intermediate 2, purple: intermediate 3, yellow: stiff.

Subsequently, to determine the PEG and dextran concentrations in their respective phases at equilibrium, six ATPS compositions were prepared for gravimetric phase separation studies, and the weights of the PEG-rich top phases were measured. These data were then used to solve a system of equations (eqs S1–S7) including mass balance equations and the equation for the binodal (Figure 3) and construct tie-lines. Specific values for empirically determined tie-lines are organized in Table 1. Monte Carlo simulations were also conducted to estimate the error associated with the tie-lines (Table S2) and are in good agreement with the numerical solutions.

The information in the completed phase diagram was used to test the hypothesis that microgel material properties correlate with the equilibrium PEG concentration in the PEG-rich phase. This hypothesis relies on the assumption that once the ATPS is well mixed, polymerization occurs within emulsions of the polymerizing species. Thus, mechanical perturbation of the ATPS is assumed to not alter the compositions of the disperse and continuous phases relative to those at equilibrium. Five ATPS compositions, corresponding to the colored data markers in Figure 3, were selected for microgel synthesis to test this hypothesis. The ATPS compositions lay on three different tie-lines, suggesting that the material properties of the microgel products would be relatively soft (red data marker), intermediate (purple, blue, and green data markers), and stiff (yellow data marker). Moreover, three ATPS compositions lay on the same tie-line, which is suggestive of intermediate material properties. These ATPS compositions are expected to separate into phases of similar compositions, but the volume ratio of their phases is expected to differ. Others have previously found that decreasing the volume ratio of the continuous phase to the disperse phase in PEG-dextran ATPSs yields larger microgel products.^22,23^ Thus, the intermediate microgel formulations are expected to have similar polymer concentrations and cross-link densities, but the intermediate 3 microgels (I.3, purple data marker) are expected to be larger than the intermediate 1 microgels (I.1, blue data marker), which are in turn expected to be larger than the intermediate 2 microgels (I.2, green data marker). The compositions and expected material properties are also outlined in Table 2.

**Figure 4:**
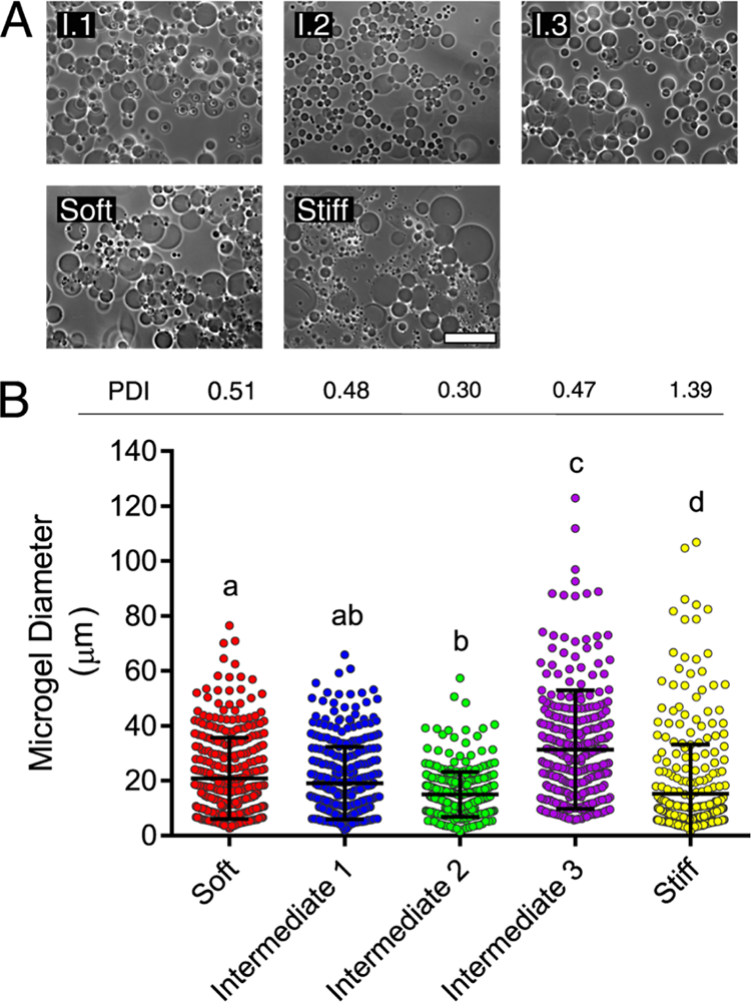
Microgel sizing analysis (A) Microgels were imaged by phase contrast microscopy. The scale bar is 100 μm. (B) Microgel diameters were manually measured in ImageJ. Error bars are standard deviations. Groups were compared by Kruskal–Wallis with Dunn’s post hoc analysis.

Microgels were synthesized according to the ATPS formulations shown in Table 2. The PEG microgels were cross-linked with an enzymatically degradable oligopeptide sequence using thiol-norbornene click chemistry (Figure 1). CGRGDS integrin binding peptide was also included, as PEG microgels are often functionalized with integrin binding peptides to facilitate cell attachment in biomedical applications.^4,5,33−35^ It is possible that the addition of these components may influence phase separation dynamics, but their molecular weights and concentrations in the ATPSs were small compared to that of the polymers. Thus, their addition to the ATPSs was assumed to be negligible. After cross-linking, the microgels were imaged under phase contrast microscopy (Figure 4A), and their diameters were measured in ImageJ. Notably, the trend of increasing microgel size with decreasing continuous phase to disperse phase volume ratio that was previously noted by Stenekes et al.^22,23^ was also observed in the intermediate microgel formulations, as expected. Intermediate 3 microgels were synthesized at the smallest dextran:PEG volume ratio and were significantly larger than intermediate 1 and intermediate 2 microgels. Intermediate 1 microgels were synthesized at a lower volume ratio than intermediate 2 microgels and are correspondingly larger, although the difference in microgel diameters was not statistically significant (Figure 4B). Interestingly, the stiff microgel formulation was highly polydisperse relative to the other microgel formulations (Figure 4B).

**Figure 5:**
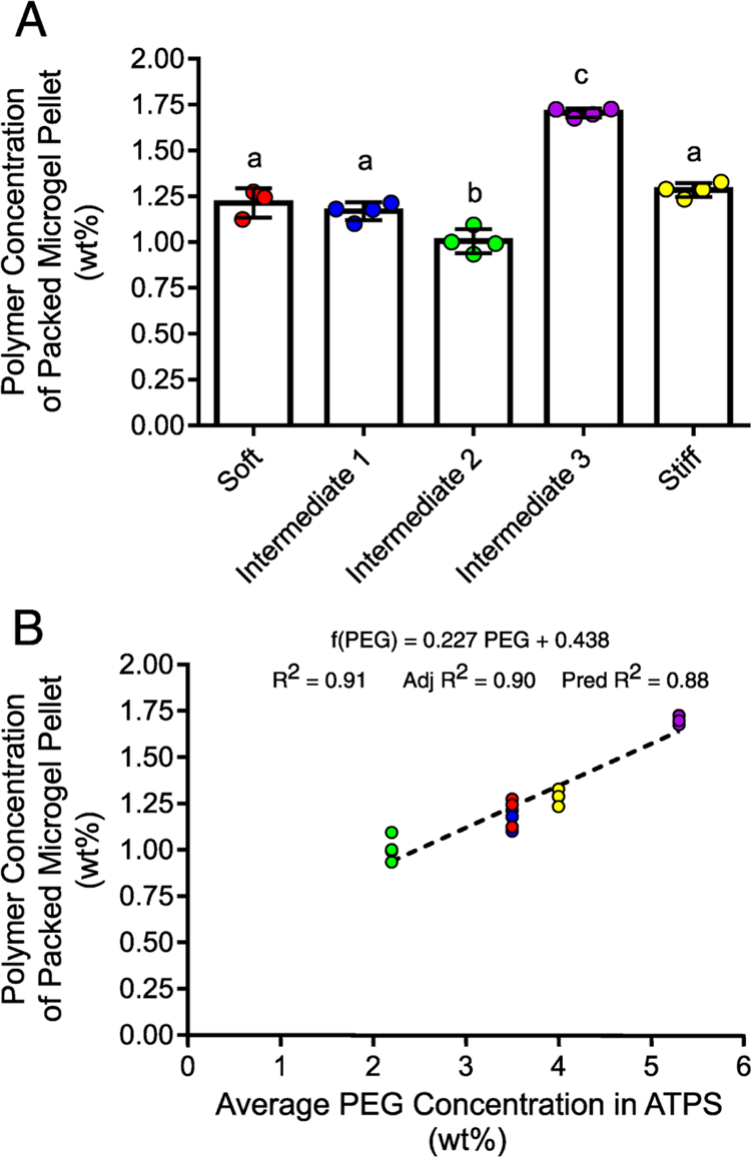
TGA of packed microgel pellets. (A) Polymer concentrations of the packed microgel pellets were determined from the TGA thermograms. Groups were compared by one-way ANOVA with Tukey’s post hoc analysis. Error bars are standard deviations. (B) Polymer concentrations of the packed microgel pellets were related to the average PEG concentrations in the ATPSs used in microgel syntheses. Data marker colors correspond to microgel formulations as follows: Red: soft, blue: intermediate 1, green: intermediate 2, purple: intermediate 3, yellow: stiff.

In order to determine the polymer concentration of the microgel formulations, microgels that were swollen in DI water were packed by centrifugation and subjected to TGA (Figure 5A). The weights of the polymer in the microgel pellets ranged from about 1.00 to 1.75 wt %. Importantly, the polymer concentrations of the microgel pellets were not related to the PEG concentrations predicted from the phase diagram. Instead, the intermediate 2 microgel formulation displayed the least polymer concentration, followed by the soft and intermediate 1 microgel formulations, which were relatively similar in polymer concentration. The stiff microgel formulation was incrementally greater in polymer concentration than the soft and intermediate 1 microgel formulations, and the intermediate 3 microgel formulation was the greatest in polymer concentration.

Interestingly, the polymer concentrations of the packed microgel pellets were linearly correlated to the average PEG concentrations in the ATPSs (Figure 5B). This finding suggests that ATPSs were sufficiently homogenized by agitation that the compositions of the separating phases during mixing are dissimilar to their compositions at thermodynamic equilibrium and are instead closer to the average composition of the ATPS. While this contradicts the assumption that microgels are polymerized within emulsions during ATPS agitation, there is compelling evidence to suggest that polymer–polymer two-phase systems can be sufficiently homogenized to undergo spinodal decomposition following simple mechanical perturbation. For example, shear-induced homogenization of polybutadiene-polystyrene-dioctylphthalate ternary solutions has been reported above critical shear thresholds. The separated phases under shear morphologically resemble strings that run parallel to the direction of shear flow. As shear rate increases, the strings become thinner until their diameters reach that of the interfacial thickness at a critical shear rate, and the system is effectively homogenized.^36,37^ There is also evidence that the structures associated with spinodal decomposition in ATPSs can be “frozen” by gelation. Lévesque et al. provided SEM micrographs of bulk microporous dextran scaffolds produced from ATPSs of PEG and dextran-methacrylate which show pore morphologies similar to the microstructures observed during spinodal decomposition.^38^ Similar porous microstructures were observed in bulk PEG hydrogels polymerized in the presence of hyaluronic acid and could be optimized to support neuron cultures.^39^ Although not explicitly mentioned by the authors, the pore morphologies of bioprinted constructs produced from methacrylated gelatin and PEG ATPS bioinks also resemble the microstructures seen in spinodal decomposition.^40^ Similar morphological phenomena have been observed in poly(acrylamide) gels polymerized in the presence of PEG, although spinodal decomposition was induced by polymerization instead of agitation of the mixture.^41^ In this regard, small-angle scattering revealed that aqueous solutions of poly(acrylamide) polymerizing in the presence of PEG demonstrate temporal profiles of scattering intensity distributions that are characteristic of spinodal decomposition.^42^ As the reactions progressed to gelation, the evolution of the intensity distributions halted, suggesting the spatial structures associated with spinodal decomposition were frozen by gelation.^42^ Structures resembling those in spinodal decomposition are also observed in polymer blends that have been quenched, in the case of temperature-induced decomposition, or cured, in the case of reaction-induced decomposition.^43^ Taken together, these observations could indicate that the PEG-dextran ATPSs used here are partially or completely homogenized by agitation such that spinodal decomposition occurs while the gelation reaction proceeds.

**Figure 6:**
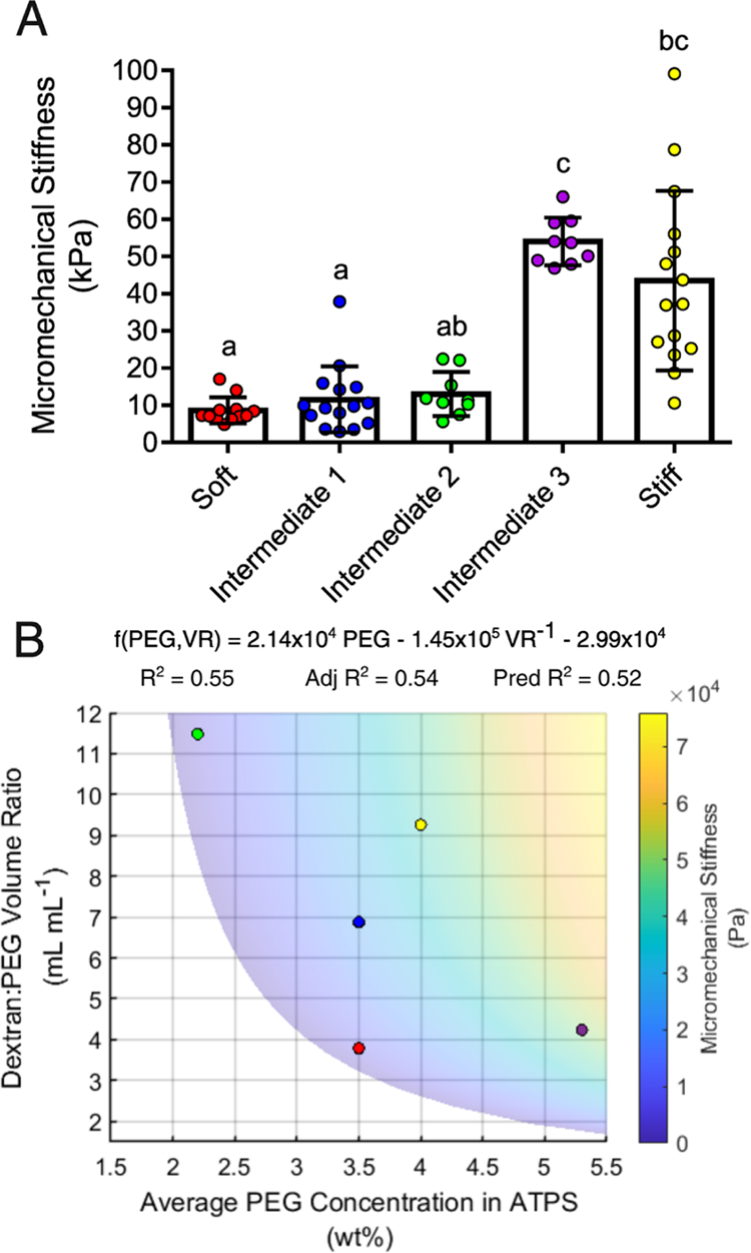
AFM of individual microgels. (A) Micromechanical stiffnesses of the microgels were determined from AFM force maps. Groups were compared by Kruskal–Wallis with Dunn’s post hoc analysis. Error bars are standard deviations. (B) Micromechanical stiffnesses were related to the average PEG concentrations in the ATPS and the dextran:PEG volume ratios (eq 2). Adj *R*^2^ and Pred *R*^2^ are the adjusted and predicted coefficients of determination, respectively. Please see Movie S2 for a 3D representation of the model surface. Data marker colors correspond to microgel formulations as follows: Red: soft, blue: intermediate 1, green: intermediate 2, purple: intermediate 3, yellow: stiff.

The microgels were also characterized by AFM to evaluate the influence of ATPS composition on the mechanical properties of microgels (Figure 6A). Similar to the findings from TGA, the PEG concentrations predicted from the phase diagram were not related to the micromechanical stiffnesses of the microgels. However, unlike the TGA data, the average PEG concentrations in the ATPS were poorly correlated to the micromechanical stiffnesses of the microgels (Figure S3). Interestingly, the data model was substantially improved by including the dextran:PEG volume ratio, as determined in the phase diagram, as an additional term (Figure 6B, Movie S2). For these data, a new phenomenological model for predicting microgel material properties is proposed, which relies on the average PEG concentration of the ATPS and the dextran:PEG volume ratio (eq 2), where PEG is the average concentration of PEG in the ATPS, VR is the volume ratio, and *a*, *b*, and *c* are fit parameters. When applied to the TGA data, eq 2 does not substantially improve the fit of the model (Figure S2 and Movie S1).
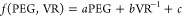
2

The coefficient of determination for the fit of the AFM data to eq 2 is still poor at *R*^2^ = 0.55 (Figure 6A), likely due to the high variances in microgel stiffness within each formulation. This might be explained by the microscale fluctuations in polymer concentrations that characterize spinodal decomposition and likely result in high variances in microscale cross-link densities upon gelation. To account for high variances in microscale material properties, microgels were assembled into bulk scaffolds using a 4-arm PEG-SH annealing linker and characterized by rheology.

**Figure 7:**
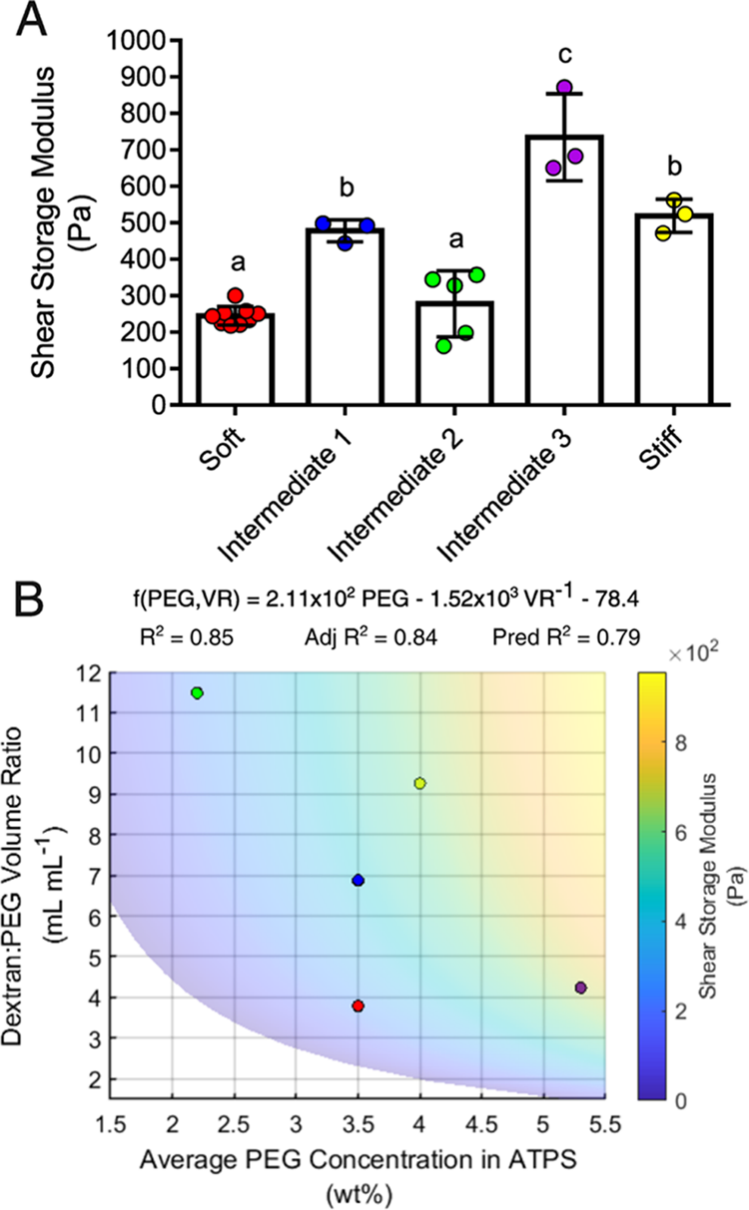
Rheology of bulk microgel scaffolds. (A) Shear storage moduli of bulk microgel scaffolds were determined from frequency sweeps. Groups were compared by one-way ANOVA with Tukey’s post hoc analysis. Error bars are standard deviations. (B) Shear storage moduli were related to the average PEG concentrations in ATPS and the dextran:PEG volume ratios (eq 2). Adj *R*^2^ and Pred *R*^2^ are the adjusted and predicted coefficients of determination respectively. See Movie S3 for a 3D representation of the model surface. Data marker colors correspond to microgel formulations as follows: Red: soft, blue: intermediate 1, green: intermediate 2, purple: intermediate 3, yellow: stiff.

The average shear storage moduli of the scaffolds assembled from the various microgel formulations were determined through frequency sweeps (Figures 7A and S4). Shear strain sweeps were also performed to ensure that frequency sweeps were conducted in the linear viscoelastic regime (Figure S5). Similar to the AFM data, the shear storage moduli of the scaffolds could be linearly correlated with the average PEG concentration in the ATPS (Figure S6), but the fit was substantially improved by modeling the data according to eq 2 (Figure 7B, Movie S3).

Previously, our lab found that the micromechanical properties of microgels used in scaffold assembly were a determinant of the scaffold’s bulk mechanical properties, as would be expected.^5^ The AFM and rheology experiments presented herein support this previous observation. Generally, the stiffest microgel formulations as determined by AFM were assembled into the stiffest scaffolds as determined by rheology (Figures 6A and 7A).

It is interesting to note that different phenomenological models were necessary to describe the TGA data set (Figure 5B) and the AFM and rheology data sets (Figures 6B and 7B). This suggests that the cross-link densities of the microgel networks are not wholly dependent upon the polymer concentrations of the microgel pellets. One possibility is cross-linker partitioning. Parlato et al. observed that simply changing the concentration of polymers and dithiothreitol cross-linker in PEG-dextran ATPSs could influence the degradation profile of PEG microgels that were susceptible to hydrolysis.^27^ Microgels that swelled to a greater extent in solvent were assumed to have lower cross-link densities and thus degraded faster by hydrolysis. They speculated that changes in the partitioning of the polymers and cross-linker in the ATPS influenced the cross-link densities of the hydrogel networks.^27^ In the work presented above, PEG is apparently homogenized sufficiently in the ATPS that its average concentration may be related to the polymer concentrations of the packed microgel pellets (Figure 5B). However, there may be heterogeneous concentrations of the peptide cross-linker within the ATPS during mixing. Cross-linker partitioning during mixing may rely on the composition of the ATPS and may occur in such a manner that reaction inefficiencies or network imperfections arise. Between these two explanations, reaction inefficiencies are less likely because cystine-containing peptides are typically efficiently incorporated in microgels synthesized from PEG-dextran ATPSs by thiol-ene chemistries.^24^ Either way, the effective cross-link density of the microgels may be reduced by cross-linker partitioning.

The TGA, AFM, and rheology data sets show that microgel material properties apparently emerge from the homogenization of the ATPS. Here, microgels were polymerized immediately after agitation during relatively early stages of decomposition. As spinodal decomposition progresses with time, the ATPS intuitively becomes less homogeneous, and how this influences microgel polymer concentration and mechanical properties is poorly understood. However, it may be possible that the Cahn–Hilliard equation, which describes the kinetics of spinodal decomposition, is an appropriate mechanistic model to predict the material properties of microgels synthesized by polymer–polymer ATPSs.^44,45^ In particular, mechanistic models of the advective Cahn–Hilliard equation may be useful.^46,47^

In order to understand the influence of phase separation kinetics on microgel material properties, the decomposition of the formulation intermediate 1 ATPS was viewed by light microscopy (Movie S4). Small droplets were distinguished within about 30 s following homogenization. By 2 min, the droplets had grown to hundreds of micrometers in size. Between 2 and 13 min, the droplets underwent flocculation to spontaneously arrange themselves into a PEG-rich droplet domain and a dextran-rich domain. Interestingly, these domains morphologically resembled bicontinuous structures that arise during the early stages of spinodal decomposition in symmetric mixtures. From 13 to 20 min, droplets continued to grow by coalescence. During this time, droplets also began to rupture, which continued up to 60 min. Few emulsions remained at 60 min, and the dextran-rich phase occupied the majority of the ATPS volume.

To understand how the kinetics of phase separation influence microgel material properties, the formulation intermediate 1 ATPS was homogenized and phase separation was allowed to proceed without further input for 5 min. The ATPS was then exposed to 365 nm light from a handheld UV flashlight for 5 min, and the ATPS was recorded for an additional 10 min after UV exposure was halted (Movie S5). During the first 5 min following agitation, phase separation proceeded in a similar fashion to what was observed in Movie S4. When the ATPS was exposed to UV light starting at 5 min, there was a short delay followed by growth of a granular mass. The mass appeared to originate from the dextran-rich continuous phase in the ATPS. The PEG droplets occasionally underwent coalescence during UV exposure, suggesting they remained liquid and were not gelled. Moreover, as the granular mass grew, the rate at which the surrounding droplets ruptured increased. Thus, polymerization appears to occur either within the continuous phase or at the droplet interface at 5 min following mixing. This further supports the concept of the peptide cross-linker partitioning into the dextran-rich phase.^48,49^ No changes in ATPS were noted following UV exposure. Although the PEG-rich droplets were hundreds of microns in size when polymerization was initiated, the granular mass was composed of smaller microgels that were tens of microns in diameter (Figure 4).

How microgel properties were influenced by the amount of time that an ATPS was allowed to coarsen before polymerization was initiated was then investigated. Formulation intermediate 1 microgels that were polymerized immediately, 20, 35, or 60 min following mixing were subjected to TGA (Figure S7). No significant differences in the polymer concentration of the microgel pellets could be detected between the immediately and 20 min polymerized microgels. However, by 35 min, the concentration of the pellet increased significantly from about 1.4 to 2.1 wt %. By 60 min, the concentration had increased to 2.6 wt %, although replicate data could not be collected for this time point because the pellet that formed was miniscule. Altogether, these data suggest later stages of ATPS coarsening result in greater packed microgel pellet polymer concentrations, which could indicate that there is a higher concentration of PEG during the later stages of coarsening where polymerization is occurring.

These data suggest that the kinetics of phase separation profoundly influence the material properties of the microgel products. Moreover, the formation and coarsening of droplets and cross-linker partitioning are likely important in microgel polymerizations conducted during later stages of ATPS separation. Therefore, mechanistic models that describe ATPS coarsening by coalescence and Ostwald ripening as well as models describing interfacial polymerization mechanisms could be useful for describing microgel properties. Further, time-course studies in which ATPSs of the same composition are mixed and then allowed to phase-separate over the course of different periods before polymerization could be helpful in attempting to relate mechanistic models to microgel material properties. King et al. have conducted similar studies to investigate the influence of separation time on the size of microgels polymerized from PEG-calmodulin conjugates in the presence of raffinose, but other material properties were not investigated.^50^ Interestingly, however, they noticed that microgel size decreased and polydispersity increased with increasing separation time, suggesting coarsening by Ostwald ripening.^50^ This same explanation likely applies to the relatively high polydispersity of microgel diameters observed in the stiff microgel formulation (Figure 3). The stiff microgel formulation is composed of the highest PEG and dextran concentrations of all of the microgel formulations, so phase separation and coarsening likely occurred the fastest in the stiff microgel formulation. This results in microgel size distributions that resemble those observed when microgels are polymerized at later time points following agitation.^50^ The stiff microgel formulation also displayed a relatively high variance in micromechanical stiffness (Figure 4A). Moreover, an inverse relationship between micromechanical stiffness and particle diameter was observed within the microgel formulations (Figure S8). Thus, the high variance in the size of the stiff formulation microgels could correspond with the high variance in their micromechanical properties and may be further explored in the future. These observations tentatively suggest that the kinetics of spinodal decomposition may broadly influence the material properties of the microgels, including both size and micromechanical properties, but further investigations are necessary.

## Conclusions

The data presented herein suggests that mixing-induced homogenization of a PEG-dextran ATPS principally influences the polymer concentration and cross-link density of its PEG microgel products during the early stages of phase separation. The dextran-rich phase-to-PEG-rich phase volume ratio, as determined from the phase diagram, was also inversely related to the micromechanical properties of the microgels and the mechanical properties of bulk microgel scaffolds, possibly due to partitioning of the peptide cross-linker. The kinetics of spinodal decomposition following ATPS homogenization may also have a profound impact on the material properties of microgel products and should be thoroughly investigated in the future.

## Supporting Information Available

NMR of PEG-amide-norbornene; determination of Tie lines; estimation of thiol-norbornene ratio for scaffold assembly; phenomenological modeling of TGA data to equation 2; phenomenological modeling of AFM data to equation 1; rheological sweeps conducted on microgel scaffolds; phenomenological modeling of rheology data to equation 1; influence of coarsening time on packed pellet polymer concentration; association between microgel size and micromechanical stiffness; Monte Carlo simulations and estimated propagated error; variance-covariance matrices for regression model; Figures S1–S8; Tables S1–S8 (PDF)
3D representation of the TGA data and model surface presented in Figure S2 (Movie S1) (MOV)
3D representation of the AFM data and model surface presented in Figure 6B (MOV)
3D representation of the bulk microgel scaffold rheology data and model surface presented in Figure 7B (Movie S3) (MOV)
Light microscopy recording of the phase separation process in the formulation intermediate 1 ATPS (Movie S4) (MOV)
Light microscopy recording of the formulation intermediate 1 ATPS undergoing polymerization (Movie S5) (MOV)

## Supplementary Material

ma3c00557_si_001.pdf

ma3c00557_si_002.mov

ma3c00557_si_003.mov

ma3c00557_si_004.mov

ma3c00557_si_005.mov

ma3c00557_si_006.mov

## Abbreviations

ATPS: aqueous two-phase system
PEG: poly(ethylene glycol)
W/O: water-in-oil
VEGF: vascular endothelial growth factor
PEGaNB: poly(ethylene glycol) amide norbornene
NMR: nuclear magnetic resonance
PEG-SH: poly(ethylene glycol) thiol
PBS: phosphate-buffered saline
LAP: lithium acyl phosphinate
I.1: intermediate 1
I.2: intermediate 2
I.3: intermediate 3
PDI: polydispersity index
TGA: thermogravimetric analysis
AFM: atomic force microscopy
SEM: scanning electron microscopy
Adj *R*^2^: adjusted coefficient of determination
Pred *R*^2^: predicted coefficient of determination
VR: volume ratio
