# Supplementary material for: Microparticle Hydrogel Material Properties Emerge from Mixing-Induced Homogenization in a Poly(ethylene glycol) and Dextran Aqueous Two-Phase System
Source: Macromolecules. 2023 Oct 30;56(21):8518–28. doi: 10.1021/acs.macromol.3c00557 (PMC10863057; doi:10.1021/acs.macromol.3c00557)
Supplement: Supplementary file 1 — ma3c00557_si_001.pdf [file ma3c00557_si_001.pdf]

Supporting Information for "Microparticle Hydrogel Material Properties Emerge from Mixing Induced Homogenization in a Poly(Ethylene Glycol) and Dextran Aqueous Two Phase System"

Thomas J. Tigner,[1] Grant Scull,[2,3] Ashley C. Brown,[2,3] Daniel L. Alge[1,4]*

1. Texas A&M University, College of Engineering, Department of Biomedical Engineering, College Station, Texas 77843, USA
2. North Carolina State University and University of North Carolina at Chapel Hill, College of Engineering, Joint Department of Biomedical Engineering, Raleigh, North Carolina 27695, USA
3. North Carolina State University, Comparative Medicine Institute, Raleigh 27695, North Carolina, USA
4. Texas A&M University, College of Engineering, Department of Material Science and Engineering, College Station, Texas 77843, USA

Email: dalge@tamu.edu

1. NMR of PEG-amide-norbornene

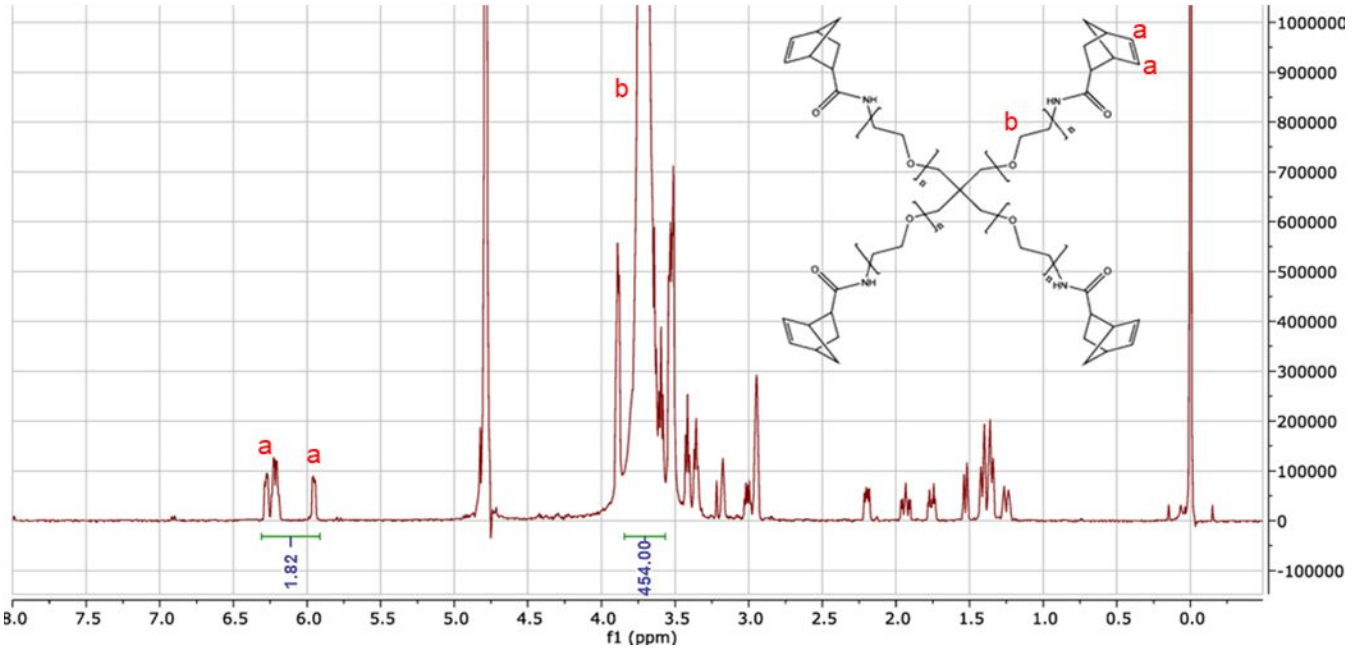

Figure S1: Representative ^{1}H NMR spectrum of PEG-amide-norbornene. The peaks that correspond to alkene protons in norbornene from 5.91-6.31 ppm were normalized to the peak that correspond to protons in the PEG backbone from 3.57-3.84 ppm, which was 454 H for one arm of the polymer. Based on these integrations, functionalization was calculated to be 91%.

2. Determination of Tie Lines

Tie-lines were calculated from the following system of mass balance equations. Where m denotes total mass in mg, and X, Y, and Z denote concentrations in wt% of PEG, dextran, and PBS, respectively. In all variables, the subscripts mix, top, and bot refer the variable to the total mixture, the top phase, and the bottom phase, respectively.

$$m_{mix} = m_{top} + m_{bot} \quad \text{(Equation S1)}$$

$$X_{top} + Y_{top} + Z_{top} = 100 \quad \text{(Equation S2)}$$

$$X_{bot} + Y_{bot} + Z_{bot} = 100 \quad \text{(Equation S3)}$$

$$X_{mix}m_{mix} = X_{top}m_{top} + X_{bot}m_{bot} \quad \text{(Equation S4)}$$

$$Z_{mix}m_{mix} = Z_{top}m_{top} + Z_{bot}m_{bot} \quad \text{(Equation S5)}$$

Moreover, the fit for the binodal has previously been determined, and is described by the function, f(x) (Equation 1). The binodal relates the concentration of dextran in either separated phase to the concentration of PEG in that same phase. Thus, plugging in concentrations of dextran into Equation 1 provides two more equations, as shown below, giving a system of seven equations total.

$$Y_{top} = f(X_{top}) \quad \text{(Equation S6)}$$

$$Y_{bot} = f(X_{bot}) \quad \text{(Equation S7)}$$

The five known variables in the system of equations are m_{mix}, m_{top}, X_{mix}, Y_{mix}, and Z_{mix}. The seven unknown variables are m_{bot}, X_{top}, Y_{top}, Z_{top}, X_{bot}, Y_{bot}, Z_{bot}. The number of unique equations equals the number of unknown variables, so a unique solution exists. The system of equations was solved for each ATPS formulation and tie-lines were constructed from the compositions of the separated phases. Tie-lines for the microgel formulations were interpolated from the empirically determined tie-lines by arc-length continuation of the conjugate curve.

3. Estimation of Thiol-Norbornene Ratio for Scaffold Assembly

Table S1: Formulations for Microgel Scaffold Assembly

Microgel Formulation	Polymer Concentration of Packed Microgel Pellet (wt%)		Estimated Thiol-Norbornene Ratio* (mol mol^{-1})
	Average	Standard Deviation	
Soft	1.21	0.08	0.83
Intermediate 1	1.17	0.05	0.86
Intermediate 2	1.01	0.07	1.04
Intermediate 3	1.71	0.02	0.58
Stiff	1.28	0.04	0.78

*Thiol-norbornene ratios were estimated from the polymer concentration of the packed microgel pellets and assumed that the gelation reactions proceeded to nearly complete efficiency.

4. Phenomenological Modeling of TGA Data to Equation 2

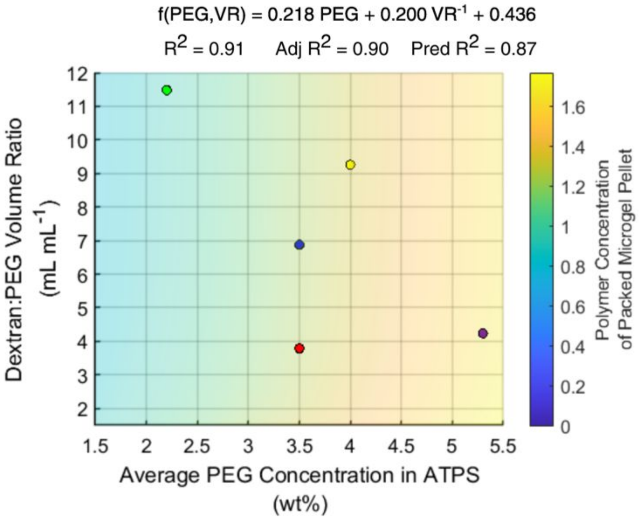

Figure S2: Relationship between ATPS composition and the polymer concentration of packed microgel pellets. The TGA data presented in Figure 5.A was related to the average PEG concentration in the ATPS and the dextran:PEG volume ratio (Equation 02). Adj R2 and Pred R2 are the adjusted and predicted coefficients of determination respectively. Please see Mov S1 for a 3D representation of the model surface. Data point color corresponds to microgel formulation as follows: Red: Soft, Blue: Intermediate 1, Green: Intermediate 2, Purple: Intermediate 3, Yellow: Stiff.

5. Phenomenological Modeling of AFM Data to Equation 1

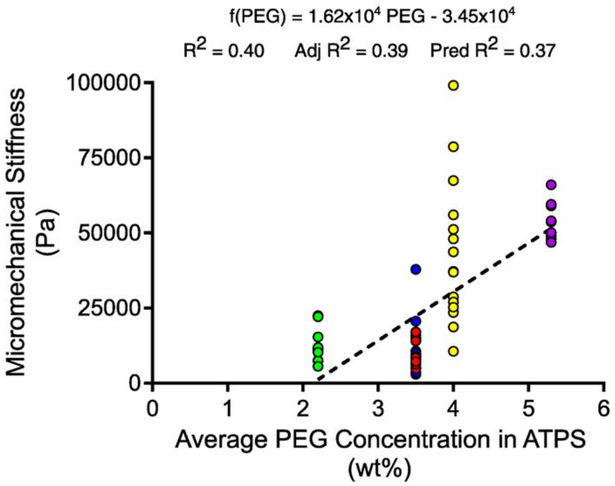

Figure S3: Relationship between ATPS composition and the micromechanical stiffnesses of individual microgels. The AFM data presented in Figure 6.A was linearly related to the average PEG concentration in the ATPS. Adj R2 and Pred R2 are the adjusted and predicted coefficients of determination respectively. Data point color corresponds to microgel formulation as follows: Red: Soft, Blue: Intermediate 1, Green: Intermediate 2, Purple: Intermediate 3, Yellow: Stiff. Average values and standard deviations for each microgel formulation may be found in Figure 6A.

6. Rheological Sweeps Conducted on Microgel Scaffolds

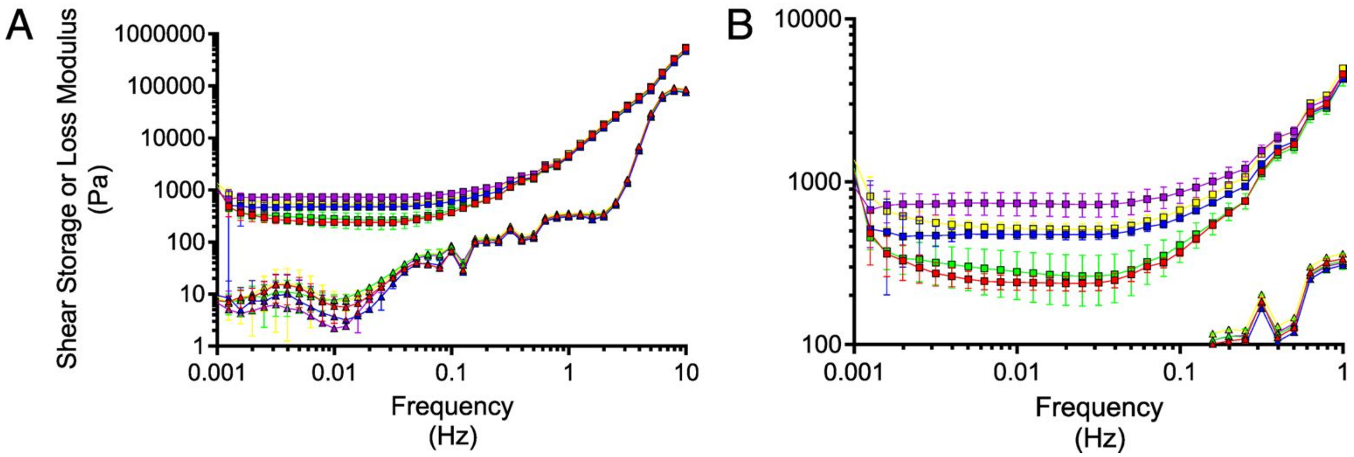

Figure S4: Microgel scaffolds were subjected to frequency sweeps. The averages of at least three frequency sweeps per microgel scaffold formulation were calculated and are plotted above. Error bars are standard deviations. Panel A and panel B are plots of the same sweeps, but the axes have been adjusted for ease of visualization. Square markers refer to storage modulus and triangle markers refer to loss modulus. Data marker colors correspond to microgel scaffold formulations as follows: Red: Soft, Blue: Intermediate 1, Green: Intermediate 2, Purple: Intermediate 3, Yellow: Stiff.

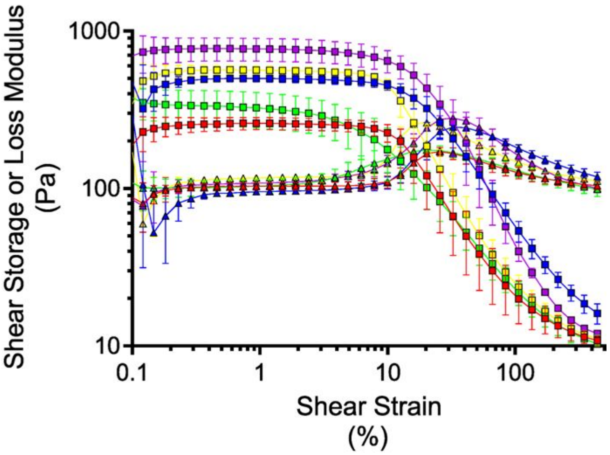

Figure S5: Microgel scaffolds were subjected to shear strain sweeps. The averages of at least three strain sweeps per microgel scaffold formulation were calculated and are plotted above. Error bars are standard deviations. Square markers refer to storage modulus and triangle markers refer to loss modulus. Data marker colors correspond to microgel scaffold formulations as follows: Red: Soft, Blue: Intermediate 1, Green: Intermediate 2, Purple: Intermediate 3, Yellow: Stiff.

7. Phenomenological Modeling of Rheology Data to Equation 1

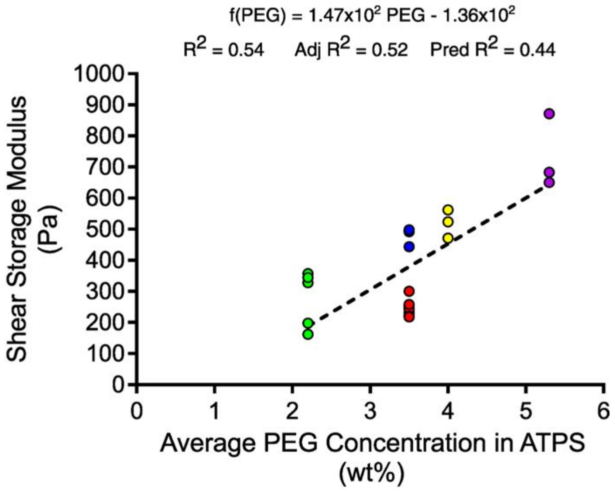

Figure S6: Relationship between ATPS composition and the shear storage modulus of bulk microgel scaffolds. The rheology data presented in Figure 7.A was linearly related to the average PEG concentration in the ATPS. Adj R2 and Pred R2 are the adjusted and predicted coefficients of determination respectively. Data point color corresponds to microgel formulation as follows: Red: Soft, Blue: Intermediate 1, Green: Intermediate 2, Purple: Intermediate 3, Yellow: Stiff. Average values and standard deviations for each microgel formulation may be found in Figure 7A.

8. Influence of Coarsening Time on Packed Pellet Polymer Concentration

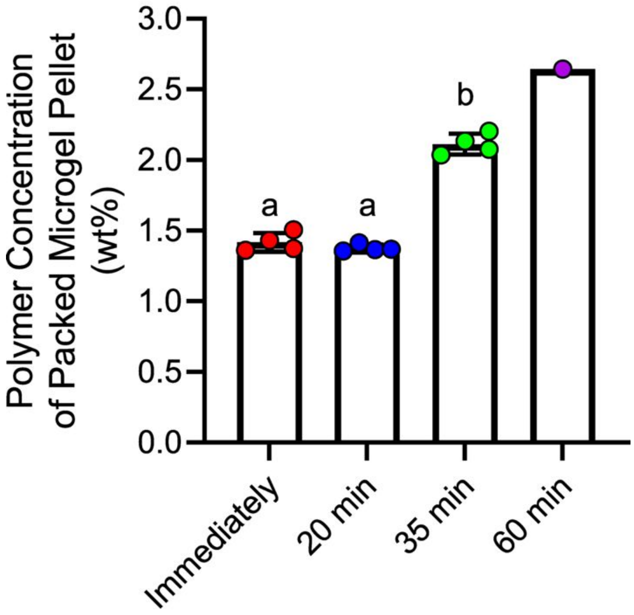

Figure S7: TGA of packed microgel pellets from formulation intermediate 1 ATPSs polymerized immediately, 20-, 35-, or 60- minutes following mixing. The polymer concentrations of the packed microgel pellets were determined from TGA thermograms. Groups were compared by One-Way ANOVA with Tukey's post hoc analysis. Error bars are standard deviations.

9. Association Between Microgel Size and Micromechanical Stiffness

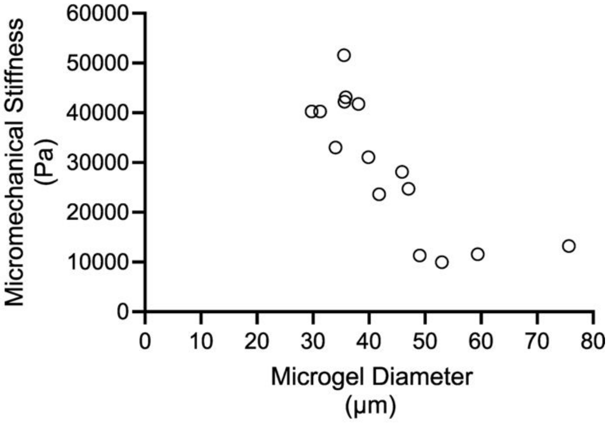

Figure S8: Relationship between the micromechanical stiffness of individual microgels and their diameters. Images were taken of formulation intermediate 3 microgels that were subjected to AFM. Stiffnesses were determined from AFM force maps and microgel diameters were measured in ImageJ.

10. Monte Carlo Simulations and Estimated Propagated Errors

Table S2: ATPS Compositions in Gravimetric Phase Separation Studies by Monte Carlo Simulation

Average PEG Concentration (wt%)	Average Dextran Concentration (wt%)	Polymer Concentrations in Top Phase* (wt%)		Polymer Concentrations in Bottom Phase* (wt%)	
		PEG	Dex	PEG	Dex
5.0	6.0	7.33 ± 0.030	0.93 ± 0.014	0.32 ± 0.016	16.17 ± 0.14
6.0	8.0	10.16 ± 0.63	0.30 ± 0.035	0.087 ± 0.071	19.42 ± 2.13
7.0	10.0	12.85 ± 0.50	0.15 ± 0.013	0.021 ± 0.012	21.78 ± 1.03
8.0	12.0	14.56 ± 0.83	0.12 ± 0.013	$7.71x10^{-4} \pm 8.38x10^{-4}$	26.56 ± 1.92
11.0	18.0	25.97 ± 1.68	$0.054 \pm 3.35x10^{-3}$	~0	31.25 ± 1.51
13.0	24.0	35.31 ± 2.14	$0.045 \pm 1.80x10^{-3}$	~0	38.00 ± 1.29

*Values are the average of the simulation ± standard deviation

Table S3: ATPS Formulation for Microgel Synthesis by Monte Carlo Simulation

Average PEG Concentration (wt%)	Average Dextran Concentration (wt%)	Polymer Concentrations in Top Phase* (wt%)		Polymer Concentrations in Bottom Phase* (wt%)	
		PEG	Dex	PEG	Dex
3.5	21.65	18.09 ± 2.22	0.086 ± 0.026	$4.74x10^{-4} \pm 4.90x10^{-4}$	26.95 ± 0.86
3.5	30.97	29.66 ± 3.78	0.050 ± 0.013	~0	35.20 ± 0.65
2.2	32.48	29.70 ± 4.24	0.050 ± 0.013	~0	35.14 ± 0.44
5.3	28.88	29.50 ± 3.32	0.050 ± 0.012	~0	35.32 ± 0.93
4.0	39.36	43.95 ± 5.65	$0.037 \pm 8.99x10^{-3}$	~0	43.38 ± 0.61

*Values are the average of the simulation ± standard deviation

Table S4: Variance-Covariance Matrix for Binodal Fit

$$f(x) = a\,e^{b\,x^{-1} + c\,x^{0.5} + d\,x^{3}}$$

Parameter		a	b	c	d
	Value	10.40	0.056	-0.43	4.32x10^{-4}
a	10.40	0.15	-4.50x10^{-3}	-7.05x10^{-3}	1.10x10^{-5}
b	0.056		1.65x10^{-4}	2.07x10^{-4}	-2.92x10^{-7}
c	-0.43			3.55x10^{-4}	-6.18x10^{-7}
d	4.32x10^{-4}				1.74x10^{-9}

Table S5: Variance-Covariance Matrix for Tie-Line Interpolation

$$f(x) = a\,x^{2} + b\,x + c$$

Parameter		a	b	c
	Value	0.022	0.085	0
a	0.022	8.79x10^{-6}	-2.62x10^{-4}	0
b	0.085		8.27x10^{-3}	0
c	0			0

Table S6: Variance-Covariance Matrix for TGA Data Model (Figure 5)

$$f(PEG) = a\,PEG + b$$

Parameter		a	b
	Value	0.227	0.438
a	0.227	3.15x10^{-4}	-1.17x10^{-3}
b	0.438		4.67x10^{-3}

Table S7: Variance-Covariance Matrix for AFM Data Model (Figure 6)

$$f(PEG,VR) = a\,PEG + b\,VR^{-1} + c$$

Parameter		a	b	c
	Value	2.14x10^{4}	-1.45x10^{5}	-2.99x10^{4}
a	2.14x10^{4}	6.52x10^{6}	-3.93x10^{7}	-1.77x10^{7}
b	-1.45x10^{5}		1.10x10^{9}	-3.52x10^{7}
c	-2.99x10^{4}			7.50x10^{7}

Table S8: Variance-Covariance Matrix for Rheology Data Model (Figure 7)

$$f(PEG,VR) = a\,PEG + b\,VR^{-1} + c$$

Parameter		a	b	c
	Value	211	-1518	-78
a	211	390	-2312	-942
b	-1518		5.50x10^{4}	-2098
c	-78			3943
